# Differential expression of basal microRNAs’ patterns in human dental pulp stem cells

**DOI:** 10.1111/jcmm.12381

**Published:** 2014-12-05

**Authors:** Punitha Vasanthan, Vijayendran Govindasamy, Nareshwaran Gnanasegaran, Wijenthiran Kunasekaran, Sabri Musa, Noor Hayaty Abu Kasim

**Affiliations:** a Department of Paediatric Dentistry and Orthodontics, Faculty of Dentistry, University of MalayaKuala Lumpur, Malaysia; b Komplex Lanai, Hygieia Innovation Sdn. BhdFederal Territory of Putrajaya, Malaysia; c Department of Conservative Dentistry, Faculty of Dentistry, University of MalayaKuala Lumpur, Malaysia

**Keywords:** medical biotechnology, gene expression, signalling network, mesenchymal stem cells

## Abstract

MicroRNAs (miRNAs) are small non-coding RNAs that regulate translation of mRNA into protein and play a crucial role for almost all biological activities. However, the identification of miRNAs from mesenchymal stem cells (MSCs), especially from dental pulp, is poorly understood. In this study, dental pulp stem cells (DPSCs) were characterized in terms of their proliferation and differentiation capacity. Furthermore, 104 known mature miRNAs were profiled by using real-time PCR. Notably, we observed 19 up-regulated miRNAs and 29 significantly down-regulated miRNAs in DPSCs in comparison with bone marrow MSCs (BM-MSCs). The 19 up-regulated miRNAs were subjected to ingenuity analysis, which were composed into 25 functional networks. We have chosen top 2 functional networks, which comprised 10 miRNA (hsa-miR-516a-3p, hsa-miR-125b-1-3p, hsa-miR-221-5p, hsa-miR-7, hsa-miR-584-5p, hsa-miR-190a, hsa-miR-106a-5p, hsa-mir-376a-5p, hsa-mir-377-5p and hsa-let-7f-2-3p). Prediction of target mRNAs and associated biological pathways regulated by each of this miRNA was carried out. We paid special attention to hsa-miR-516a-3p and hsa-miR-7-5p as these miRNAs were highly expressed upon validation with qRT-PCR analysis. We further proceeded with loss-of-function analysis with these miRNAs and we observed that hsa-miR-516a-3p knockdown induced a significant increase in the expression of WNT5A. Likewise, the knockdown of hsa-miR-7-5p increased the expression of EGFR. Nevertheless, further validation revealed the role of WNT5A as an indirect target of hsa-miR-516a-3p. These results provide new insights into the dynamic role of miRNA expression in DPSCs. In conclusion, using miRNA signatures in human as a prediction tool will enable us to elucidate the biological processes occurring in DPSCs.

## Introduction

Dental pulp stem cells (DPSCs) have emerged as a promising source of cells for numerous applications in regenerative medicine. Once thought to be seed cells only for tooth tissue regeneration, currently, these cells are being investigated for repair of tissues outside the tooth. We have shown that DPSCs are able to differentiate into myriad types of cells 1. Likewise, others have a successful outcome of using these cells in pre-clinical animal disease models 2. DPSCs are present in ‘cell-rich zones' within the dental pulp region and are considered to have similar characteristics as BM-MSCs, *e.g*., self-renewal capability and multi-lineage differentiation 3,4. Previously, we conducted a gene profiling study between DPSCs and other types of MSCs and found that although these cells shared basic MSCs criteria, they retained unique gene characteristics which make them different from one another. For instance, DPSCs are primed towards neuro-ectoderm lineages as compared with other cell lines 5. We reckoned that these phenomena are because of molecular networks and regulatory pathways. However, knowledge of these fundamental cues in DPSCs is still insufficient. Hence, optimal conditions and signals, especially involving gene expression regulation governing the fate of DPSCs, need to be identified.

One of the molecular regulatory factors that have received increasing attention is miRNAs, which have the ability to regulate many target genes and control gene expression through translational repression and degradation 6. MicroRNA (miRNAs) are ∼20–22 nucleotides in length and well known to govern a broad array of cellular functions by influencing the abundance and translation efficiency of cognate mRNA. One single miRNA can target multiple sites of genes on mRNA transcripts, and conversely, a single mRNA can be targeted by multiple miRNAs 7,8. This regulation is performed on two bases: cis regulation in which miRNA directs target mRNA and either represses their translation or regulates degradation at post-transcriptional level. On the other hand, miRNA also appears to provide a subsequent effect that may exert the level of other mRNA as well as protein interactions through trans-regulatory mechanisms 9.

They are also essential regulators that can contribute to intrinsic stem-cell (SC) properties such as self-renewal, SC pluripotency and differentiation 10. For instance, differentiation was found to be directly associated with cell cycle exit in which miRNA tends to cause negative regulation of oncogenes, which otherwise would promote proliferation 11. Moreover, they were shown to be involved in differentiation 12, controlling developmental time-point and homoeostasis through diverse cellular processes by focusing on specific pathways within cells.

Thus, in this study, expression profiling of miRNAs found in DPSCs as compared with the BM-MSCs, which is always regarded as a golden cell source in regenerative medicine, was carried out for the first time to uncover molecular signatures and regulatory pathways that could broaden our understanding of the roles of miRNAs for future experimental and clinical applications. Ultimately, this can be used as a primordial approach to comprehend the DPSCs' biological progressions to ensure success when applied in SC therapy.

## Materials and methods

### Tissue collection and isolation of cells

This study was conducted with written consents from all donors after being reviewed and approved by the Medical Ethics Committee, Faculty of Dentistry, University of Malaya [Medical Ethics Clearance Number: DF CO1107/0066(L)].

A volume of 60 ml of BM-MSCs aspirates was obtained from the iliac crest of three healthy donors (*n* + 3) under deep sedation (age: 24–35) as previously described by us 5. Briefly, the BM-MSCs were diluted (1:1) in knockout (KO) DMEM (Invitrogen, Carlsbad, CA, USA; www.invitrogen.com), and centrifuged at 706.3524 × g for 10 min. to remove anti-coagulants. After centrifugation, the mononuclear cells (MNC) were isolated by layering onto a lymphoprep density-gradient media (1:2; Axis-Shield PoC AS). The MNC present in the buffy coat were then washed with culture medium (consisting of basal media of KO-DMEM, 10% Australian characterized foetal bovine serum (FBS, Hyclone, Thermo Scientific Inc, Waltham, MA, USA, http://www.thermofisher.com), 1% Glutamax (Invitrogen) and 0.5% Penicillin/Streptomycin (Invitrogen). The mononuclear fractions that also contained SCs were plated onto culture flasks. Separately, DPSCs cultures were isolated from sound and intact third molars of adults (age: 24–35, *n* + 3) as previously described by us 13. Prior to isolation, root surfaces were cleaned with povidone-iodine (Sigma-Aldrich, St. Louis, MO, USA; http://www.sigmaaldrich.com) and the pulp was removed within 2 hrs post-extraction. The pulp tissues were minced into smaller fragments, and treated with a solution of 3 mg/ml of collagenase type I (Gibco, Grand Island, NY, USA; http://www.invitrogen.com) for 40 min. at 37°C. After inactivation with 10% FBS, the cells were then centrifuged and seeded in a conventional tissue culture flask. Similar culture conditions were provided for both cells, namely in T75 cm^2^ culture flasks (BD Pharmingen, San Diego CA, USA; http://www.bdbiosciences.com) with culture medium containing KO-DMEM, 0.5% and 10,000 μg/ml of Penicillin/Streptomycin (Invitrogen); 0.01× Glutamax (Invitrogen) and 10% FBS with humidified atmosphere of 95% air and 5% CO_2_ at 37°C. Non-adherent cells were removed after 48 hrs of initial plating by intensely washing the flask. The medium was replaced every 3 days until the cells reached 80–90% confluence.

### Growth Kinetics

Analysis of proliferation capacity was determined by plating 25,000 cells/cm^2^ of each SC into separate T75 cm^2^ culture flasks (BD Pharmingen). When the cells reach 90% confluence, they were then trypsinized. Cells were counted and evaluated for viability by means of Tryphan Blue dye exclusion before sub-culturing. Cells were re-plated for a total of 5 subsequent passages (P1–P5), with three replicates for each passage. To compare the expansion rate for both cells, the population doubling time (PDT) values were determined. The PDT was obtained by using the formula:

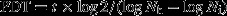


N_i_: the inoculums cell number; N_h_ is the cell harvest number and *t* is the duration of the culture (in hours).

### Cell cycle analysis

The cells were pre-seeded on a 35-mm tissue culture dish (BD Pharmigen) at a density of 5000 cells/cm^2^. Upon reaching 90% confluence, the cells were detached, fixed and permeabilized in 70% ethanol and left overnight at 4°C. Thereafter, 500 μl was extracted (containing 1 × 10^6^ cells), and DNA was stained with Propidium iodide/RNAse staining buffer (BD Pharmigen) for 15 min. at room temperature and subsequently washed in Dulbecco's PBS (DPBS; Invitrogen). DNA content was analysed on Guava Technologies (Millipore, Billerica, MA, USA) flow cytometer by using Cytosoft, Version 5.2, Guava Technologies software.

### Flow cytometric analysis

At P3, cells were harvested by trypsinization with 0.05% trypsin (Invitrogen) upon reaching 90% confluence, and re-suspended in DPBS to reach a final cell density of 1.5 × 10^6^ cells/ml. An amount of 200 μl of cell suspension (1 × 10^5^ cells) was incubated in the dark for 1 hr at 37°C with Phycoerythrin-conjugated antibodies against CD44, CD73, CD166, CD105 and CD34, and fluoro isothycyanate-conjugated antibodies against CD45 and HLA-DR (all from BD Pharmingen) for specific surface antigens analysis by using flow cytometer. Excess antibodies were removed by washing with DPBS. All analyses were standardized against negative control cells incubated with Isotype-specific IgG1-PE and IgG1-FITC (BD Pharmingen). At least 10,000 events were acquired on Guava Technologies flow cytometer, and the results were analysed by using Cytosoft, Version 5.2, Guava Technologies.

### *In vitro* tri-lineage differentiation assay

A total of 1000 of P3 cells/cm^2^ in 6-well plates were grown until confluence, and induced to multi-lineage differentiation as defined by Govindasamy *et al*. 13 with the following formulae: adipogenic differentiation medium: media supplemented with 10% FBS, 200 μM indomethacin, 0.5 mM 3-Isobutyl-l-methyxanthine, 10 μg/ml insulin and 1 μM dexamethasone (all reagents from Sigma-Aldrich); chondrogenesis differentiation medium: media supplemented with ITS+1 (Sigma-Aldrich), 50 μM of L-ascorbic acid-2 phosphates, 55 μM of sodium pyruvate (Invitrogen), 25 μM of L-proline (Sigma-Aldrich) and 10 ng/ml of a transformation growth factor-beta (TGF-β) (Sigma-Aldrich); osteogenic differentiation medium: media supplemented with 10% FBS, 10^−7^ M dexamethasone, 10 mM-glycerol phosphate (Fluka, Buchs, Switzerland) and 100 μM of L-ascorbic acid-2 phosphate.

### Evaluation of tri-lineage differentiation

After ∼21 days of differentiation, the cells were fixed for cytochemical staining. Lipid droplets were visualized by using Oil Red O staining (Sigma-Aldrich), proteoglycans accumulation was visualized by Alcian Blue staining (Sigma-Aldrich) and calcium accumulation was visualized by using Von Kossa staining (Sigma-Aldrich) for adipogenic, chondrogenic and osteogenic differentiation respectively. The cells were also analysed by using quantitative RT-PCR (qRT-PCR). Total RNA was extracted by using Trizol (Invitrogen) and reverse-transcribed into cDNA by using Superscript II reverse transcriptase (Invitrogen) according to the manufacturer's instructions. The qRT-PCR mixture contained cDNA, forward and reverse primers, and SYBR Green PCR Master Mix (Applied Biosystems, Foster City, CA, USA). The reactions were conducted by using AbiPrism 7000 Sequence Detection System (Applied Biosystems) with initial enzyme activation at 95°C for 10 min., followed by 45 cycles of denaturation at 95°C for 15 sec. and annealing and extension at 60°C for 60 sec. The expression level of genes of interest was normalized against housekeeping gene GAPDH. The fold change was calculated by using the equation 2^−ΔΔCT^. Primer sequences are presented in Table S1.

### miRNAs isolation

Culture medium was aspirated, discarded and rinsed with DPBS. Cells at P3 (*n* + 3) were then trypsinized to detach them from the flask and counted. Immediately, culture medium was added to inactivate the trypsin, and centrifuged to pellet the cells. An estimated 10^2^ to 10^3^ million cells were collected for the miRNA isolation. The foremost step carried out for the mirVana miRNA Isolation Kit procedure was to disrupt samples in a denaturing lysis buffer. Next, samples were subjected to Acid-Phenol:Chloroform extraction, which provides a robust front-end purification that also removes most DNA 14. The procedure to obtain miRNAs was according to the manufacturer's protocol (mirVana miRNA isolation kit, Ambion, Life Technologies, Austin, TX, USA).

### Profiling of miRNAs

Profile analysis of human encoded miRNAs was performed by using the TaqMan MicroRNA Assay (Applied Biosystems). Briefly, TaqMan MicroRNA Assays included two steps: stem loop reverse transcription (RT) followed by real-time quantitative PCR (90 ng/Rx with 24-multiplex primers). Each of the 10 μl RT reaction tube which included 90 ng total RNA, 50 nM stem-loop RT primers, 1× RT buffer, 1.25 mM each of dNTPs, 0.25 U/μl RNase inhibitor and 10 U/μl MultiScribe Reverse Transcriptase was incubated in a PTC-225 Peltier Thermal Cycler (MJ Research, Watertown, MA, USA) for 30 min. at 16°C and at 42°C, followed by 5 min. at 85°C and then maintained at 4°C. RT products were diluted 20 times with dH_2_O prior to the setting up of the PCR reaction. Real-time PCR for each miRNA was carried out in triplicates, and each 10 μl reaction mixture included 2 μl of diluted RT product, 5 μl of 2× TaqMan Universal PCR Master Mix and 0.2 μM TaqMan probe. The reaction tube was incubated in an Applied Biosystems 7900HT Sequence Detection System at 95°C for 10 min., followed by 40 cycles at 95°C for 15 sec. and 60°C for 1 min. The threshold cycle (C_t_) is defined as the fraction of cycle number at which the fluorescence exceeds the fixed threshold of 0.2. As an endogenous control, total RNA input was normalized based on the Ct values of the TaqMan U6 snRNA assay. The fold change was calculated as 2 – Ct × *K*, where Ct + [Ct miRNA − Ct U6snRNA] and *K* is a constant 15.

### Quantitative validation of miRNA using qRT-PCR

Quantitative reverse transcription-PCR (RT-PCR) was carried out by using 25 ng of total RNA by using the mirVana quantitative RT-PCR miRNA Detection Kit (Ambion, Life Technologies) with mirVana quantitative RT-PCR primer sets (Ambion, Life Technologies) for the 10 miRNAs of interest that are listed in Table S2. Detection of amplification was performed with SYBR green nucleic acid stain (Invitrogen) by using an Applied Biosystems-Real time Detection System. The miRNAs expression levels were calculated by using comparative cycle threshold (C_t_) method. C_t_ values of target miRNAs were normalized in relation to U6 snRNA, which is an internal control gene. The fold change was calculated by using the equation 2^−ΔΔCT^.

### Pathway analysis and prediction

Predicted miRNA targets were determined by using the miRanda algorithm (http://microrna.sanger.ac.uk/targets/v5/) and TargetScan v4.2 (http://www.targetscan.org/). Common predicted targets as well as targets from each database were subjected to pathway exploration by using the Ingenuity Pathway Analysis (IPA) software (Ingenuity Systems, Redwood City, CA, USA). An IPA (Core) Analysis is the process of mapping uploaded data to the IPA Knowledge Base (KB), and creating molecular networks by generating pathways algorithmically. This pathway was developed by dividing data into diseases and biological functions that are overrepresented in our data. To avoid exceeding the maximum gene list size allowed by the IPA program, we limited targets based on assigned score by each program. Therefore, scores of at least 17 and 20.31 were set for miRanda and TargetScan respectively. Using this software and its accompanying interactive database, the top-ranked pathways were determined based on the incidence of predicted miRNA targets in a list of canonical pathways provided by the software. IPA also produced the top-ranked networks where the predicted miRNA targets were found according to gene ontology. Additionally, the biological functions associated with these networks are also provided.

### Transient transfection of miRNA mimics and inhibitors

The miRNA mimics, inhibitors and negative controls for hsa-miR-516a-3p and hsa-miR-7-5p were purchased from (*mir*Vana^*®*^, Life Technologies™). DPSCs were transfected with the mimic, inhibitor and negative control at final concentrations of 20 nM. The siPORT NeoFX transfection agent (Ambion, Austin, TX, USA) was used according to the manufacturer's instructions. Briefly, cells were digested with 0.25% trypsin when they reached 80% confluence. The transfection agent was mixed, and incubated for 10 min. at room temperature. Cell suspension was overlaid onto the transfection complexes, and incubated at 37°C for 24 and 48 hrs for further miRNA and mRNA analysis. Transfection efficiency was determined by qRT-PCR.

### Real-time RT-PCR of mRNA expression

The transfected cells were analysed for selected target mRNA expression by using quantitative RT-PCR. Total RNA was extracted by using Trizol (Invitrogen), and was then reverse-transcribed into cDNA by using Superscript II reverse transcriptase (Invitrogen) according to the manufacturer's instructions. The qRT-PCR mixture contained cDNA, forward and reverse primers, and SYBR Green PCR Master Mix (Applied Biosystems). The reactions were conducted by using AbiPrism 7000 Sequence Detection System (Applied Biosystems) with initial enzyme activation at 95°C for 10 min., followed by 45 cycles of denaturation at 95°C for 15 sec., and annealing and extension at 60°C for 60 sec. The expression levels of wingless-type MMTV integration site family, member 5A (WNT5A) and epidermal growth factor receptor (EGFR) were normalized against the housekeeping gene alpha-tubulin. The relative expression levels were normalized against Human cDNAs (Positive control), which had also been normalized to 1. The fold change was calculated by using the equation 2^−ΔΔCT^. Primer sequences are presented in Table S1.

### Western blot analysis

Western blot analysis was performed after the whole transfected cell lysate was extracted by using Cytobuster (Novagen, Milipore, Billerica, MA, USA) lysis buffer. The cell lysate was then treated with protease inhibitor cocktail (Milipore). Prior to loading on to gel, protein quantification was carried out against bovine serum albumin (ThermoScientific, Wilmington, DE, USA) by using Bradford method. The proteins were loaded on 10% sodium dodecyl sulphate-polyacrylamide gels, and then transferred to polyvinylidene fluoride membranes. Blocking and washing were performed according to the manufacturer's instructions (Western blot kit, Pierce ECL, ThermoScientific). The membranes were left overnight with the following primary antibodies: rabbit anti-human WNT5A; rabbit anti-human EGFR; and alpha tubulin as control (Abcam, Cambridge, UK). Thereafter, the membranes were incubated with peroxidase-conjugated secondary antibody (Abcam). The blots were visualized by using a chemiluminescence detection system.

### Reporter vectors and luciferase assay

The oligonucleotides of the putative hsa-miR-516a-3p recognition element, at the nucleotides of 1650-1656 of the 3′-untranslated region (3′-UTR) of the human WNT5A gene wild-type, were designed by using human genomic DNA with flanking Pst1 and EcoRV sites (forward: 5′-*CTGCAG*TCCAGTTGGGATTATTC-3′ and 5′-*GATATC*TTCAACCCAACACGC-3′). Meanwhile, the mutant type was constructed by deleting 3 nucleotides of the seed region with the flanking EcoRV and HindIII sites (forward: 5′-*GATATC*TCAAAGTATTTTGTAC-3′ and 5′-*AAGCTT*CCTCAGAAACAAGG-3′). After annealing the sense and anti-sense oligonucleotides, the DNA fragment products were double digested by using the above-indicated restriction sites and cloned into pSV40-CLuc (New England Biolabs, Ipswich, MA, USA) vector. The resulting vector wild-type indicated as pSV40-WNT5A-WT or mutant type as pSV40-WNT5A-MT was then transfected by using Lipofectamine 2000 (Invitrogen) into hsa-miR-516a-3p mimics (miR-516) DPSCs (*mir*Vana^*®*^, Life Technologies^™)^ or miR-negative control (miR-NC) DPSCs (2 × 10^4^ cells) seeded in a 24-well plates. After 48 hrs of incubation, the cell extracts were prepared for luciferase assay. A thymidine kinase promoter-driven secreted Gaussian luciferase (pTK-GLuc, New England Biolabs) was used as an internal control. The relative luciferase activity was calculated by normalizing transfection efficiency to the internal control. All experiments were carried out in 3 technical replicates.

### Statistical analysis

All values are given as mean and SD. Data were analysed by using the SPSS statistical software, version 19.0 (SPSS Inc, Chicago, IL, USA). The data were analysed by using two-way anova. The significance level was set at *P* + 0.05. Tukey post-hoc multiple comparisons were carried out to determine the differences between the groups. Mean ± SD values are shown from either three independent biological or technical experiments.

## Results

### Elementary depiction of dental pulp and bone marrow SCs

For physiognomies interpretation, both BM-MSCs and DPSCs showed appearance of fibroblastoid cells in spindle-shaped morphology (Fig. S1A), which was retained in all subcultures. DPSCs cultures consistently displayed a higher incidence (12.7% ± 2.1%) of cells in the S + G2 + M phases of the cell cycle with ∼85% of cells in phase G1/GO when compared with BM-MSCs (5.7% ± 1.2%) of cells in S + G2 + M with ∼90% of the cells in phase G1/GO (Fig. S1B). The accumulation cell number was also compared for BM-MSCs and DPSCs throughout 5 passages (Fig. S1C). The graph for DPSCs showed a lag phase for 2 passages, and then multiplying at a rapid rate before reaching a plateau stage earlier than BM-MSCs. Furthermore, the PDT for DPSCs in P1 was 20.50 ± 1.39 hrs, whereas 27.01 ± 0.73 hrs was recorded for BM-MSCs. At P5, the PDT was 23.12 ± 0.65 hrs for DPSCs and 33.16 ± 0.97 hrs for BM-MSCs respectively (Fig. S1C). Collectively, these results show DPSCs having a higher proliferation rate as compared with BM-MSCs, conforming to our previous 5 as well as other independent studies 16. Moreover, antigenic phenotypes for both cells were examined by using flow cytometric analyses as shown in Figure S2. The results revealed that DPSCs were positive (>85%) for many markers similar to BM-MSCs: CD44, CD 73, CD90, CD105 and CD166. At the same time, DPSCs were negative (<2%) for haematopoietic surface markers, such as CD34, CD45, HLA-DR.

Cultivation of confluent DPSCs and BM-MSCs was then introduced to multipotent differentiation (Fig. S3A). Accumulation of neutral lipid vacuoles indicated by the Oil Red O stain revealed adipogenic differentiation in both cell lines. However, the observation showed larger and dense lipid vacuoles (red colour) in BM-MSCs compared with DPSCs, which were smaller and scattered remotely throughout the flask. Similar effects were seen when there was a higher exposure of dark-stained mineralized matrix in BM-MSCs in comparison with DPSCs, which indicates efficient osteogenic differentiation. Chondrogenic differentiation was confirmed with the presence of proteoglycan by using Alcian Blue in both cell lines. No staining was seen in undifferentiated cells; however the data are not shown here. Besides that, the cells also showed mRNA expression of runt-related transcription 2, osteocalcin, peroxisome proliferation activated receptor 2 lipoprotein lipase, aggrecan and collagen 2A1 (COL2A1). These findings are typical for osteoblast cells, adipocytes and chondrocytes (Fig. S3B).

### Differential expression of miRNAs between dental pulp and bone marrow SCs

**Fig 1:**
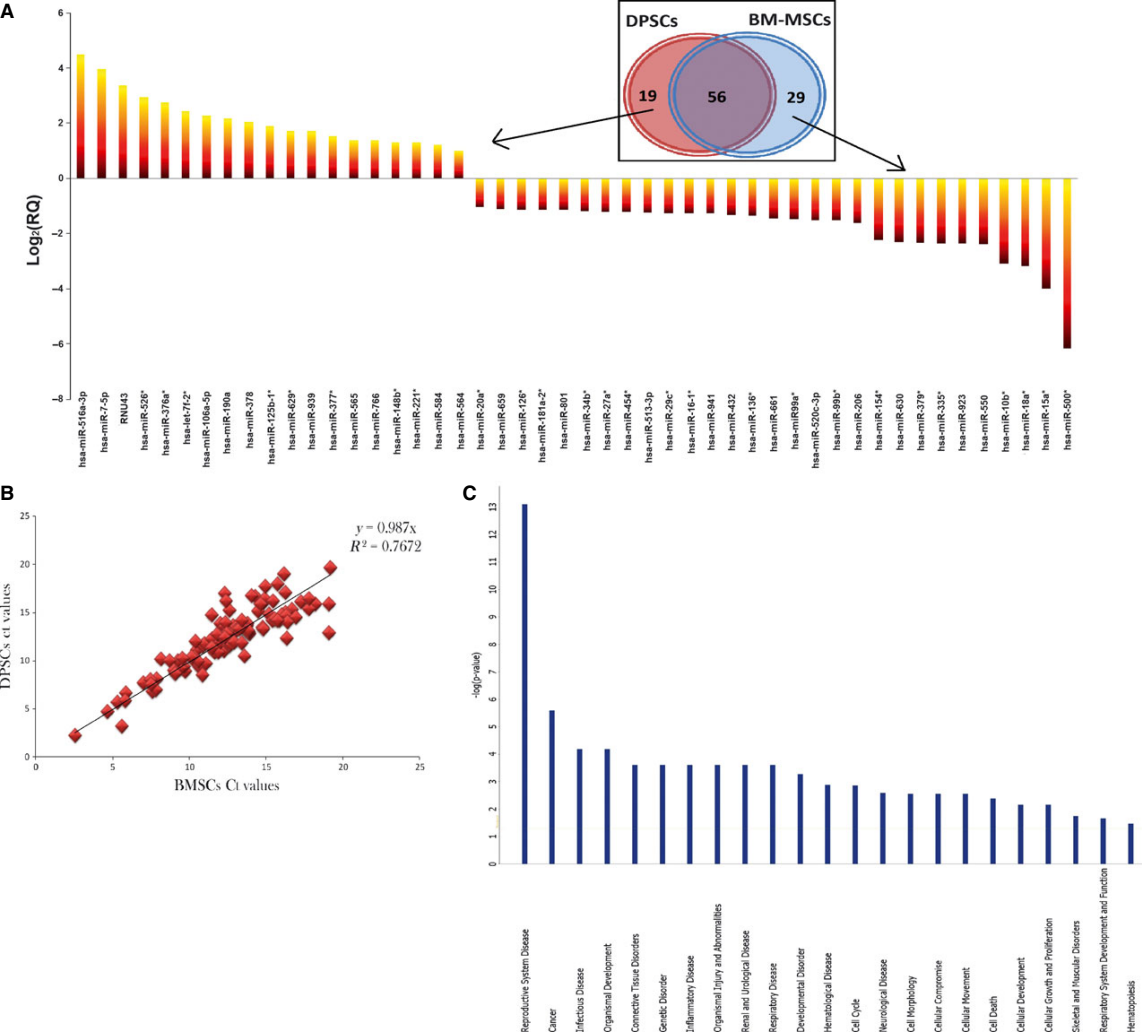
miRNAs manifestation in DPSCs compared with BM-MSCs. (A) The log_2_ of RQ value was used to plot the relative fold change. *Y*-axis: log_2_ RQ, *X*-axis: miRNA. Sorted Log_2_ RQ shows 29 miRNAs with decreased expression and 19 with increased expression in DPSCs. The most significant difference was seen in hsa-miR-500* with decreased expression, and hsa-miR-516a-3p with increased expression [RQ + 2^−ΔΔCt^, ΔΔCt + ΔCt (DPSCs) − ΔCt (BM-MSCs), ΔCt + Ct (target miRNA) − Ct (endogenous control)]. Venn diagram showing the number of shared and specific miRNAs for DPSCs and BM-MSCs. (B) Scatter plot and correlation analysis between DPSCs and BM-MSCs with standard correlation found to be R2 is over 76%. (C) Major functions inclined by miRNA action on putative target genes using IPA of MSCs of BM *versus*DP. Height of bar is determined by projected involvement of the particular pathways.

Based on the analysis of the 104 miRNAs, it is clearly shown in Table1, that 48 miRNAs were differentially expressed between BM-MSCs and DPSCs. Among the differentially expressed miRNAs, 19 of them were up-regulated in DPSCs, while 29 were down-regulated. In addition, a total of 56 miRNAs (53.8%) with ΔΔCts value between +1 and −1 were shown to be commonly expressed between the two subsets of cells (Fig.1A). Furthermore, there was a high correlation of miRNA expression pattern between DPSCs and BM-MSCs, with *R*^2^ 76% (Fig.1B). The fold change value of each miRNA is presented in Table S3.

**Table 1:** Sorted Log_2_ (fold change) of 104 miRNA between DPSCs and BMSCs using ΔΔCts. 53.85% of ΔΔCts (56 of 104 determined assays), were between +1 and −1

Up-regulated	Down-regulated		Between +1/−1		
hsa-miR-516a-3p	hsa-miR-20a^*^	hsa-miR-154^*^	hsa-miR-15b^*^	hsa-miR-509-3p	hsa-miR-188-5p
hsa-miR-7-5p	hsa-miR-659	hsa-miR-630	hsa-miR-138-1^*^	hsa-miR-601	hsa-miR-214^*^
RNU43	hsa-miR-126^*^	hsa-miR-379^*^	hsa-miR-149^*^	hsa-miR-543	hsa-miR-432^*^
hsa-miR-526b^*^	hsa-miR-181a-2^*^	hsa-miR-335^*^	hsa-miR-151-3p	hsa-miR-589^*^	hsa-miR-130b^*^
hsa-miR-376a^*^	hsa-miR-801	hsa-miR-923	hsa-miR-19b-1^*^	hsa-miR-625^*^	RNU48
hsa-let-7f-2-3p	hsa-miR-34b^*^	hsa-miR-550	hsa-miR-27b^*^	hsa-miR-638	hsa-miR-93^*^
hsa-miR-106a	hsa-miR-27a^*^	hsa-miR-10b^*^	hsa-miR-22^*^	hsa-miR-643	hsa-miR-7-1^*^
hsa-miR-190a	hsa-miR-454^*^	hsa-miR-18a^*^	hsa-miR-26a-1^*^	hsa-miR-656	hsa-miR-505^*^
hsa-miR-378	hsa-miR-513-3p	hsa-miR-15a^*^	hsa-miR-26b^*^	hsa-miR-769-5p	hsa-miR-181a^*^
hsa-miR-125b-1^*^	hsa-miR-29c^*^	hsa-miR-500^*^	hsa-miR-30e^*^	hsa-miR-877	hsa-miR-222^*^
hsa-miR-629^*^	hsa-miR-16-1^*^		hsa-miR-30a^*^	hsa-miR-942	hsa-miR-135a^*^
hsa-miR-939	hsa-miR-941		hsa-miR-30d	RNU24	hsa-miR-493^*^
hsa-miR-377^*^	hsa-miR-432		hsa-miR-30e	RNU44	hsa-miR-145^*^
hsa-miR-565	hsa-miR-136^*^		hsa-miR-34a^*^	RNU6B	hsa-miR-875-5p
hsa-miR-766	hsa-miR-661		hsa-miR-411^*^	hsa-miR-768-3p	hsa-miR-30d^*^
hsa-miR-148b^*^	hsa-miR-99a^*^		hsa-miR-409-3p	hsa-miR-373^*^	hsa-miR-550^*^
hsa-miR-221^*^	hsa-miR-520c-3p		hsa-miR-424^*^	hsa-let-7i^*^	hsa-miR-21^*^
hsa-miR-584	hsa-miR-99b^*^		hsa-miR-425^*^	hsa-miR-100^*^	hsa-miR-760
hsa-miR-564	hsa-miR-206		hsa-miR-770-5p	hsa-miR-30a	

### Analysis of up-regulated miRNAs expressed in DPSCs

**Fig 2:**
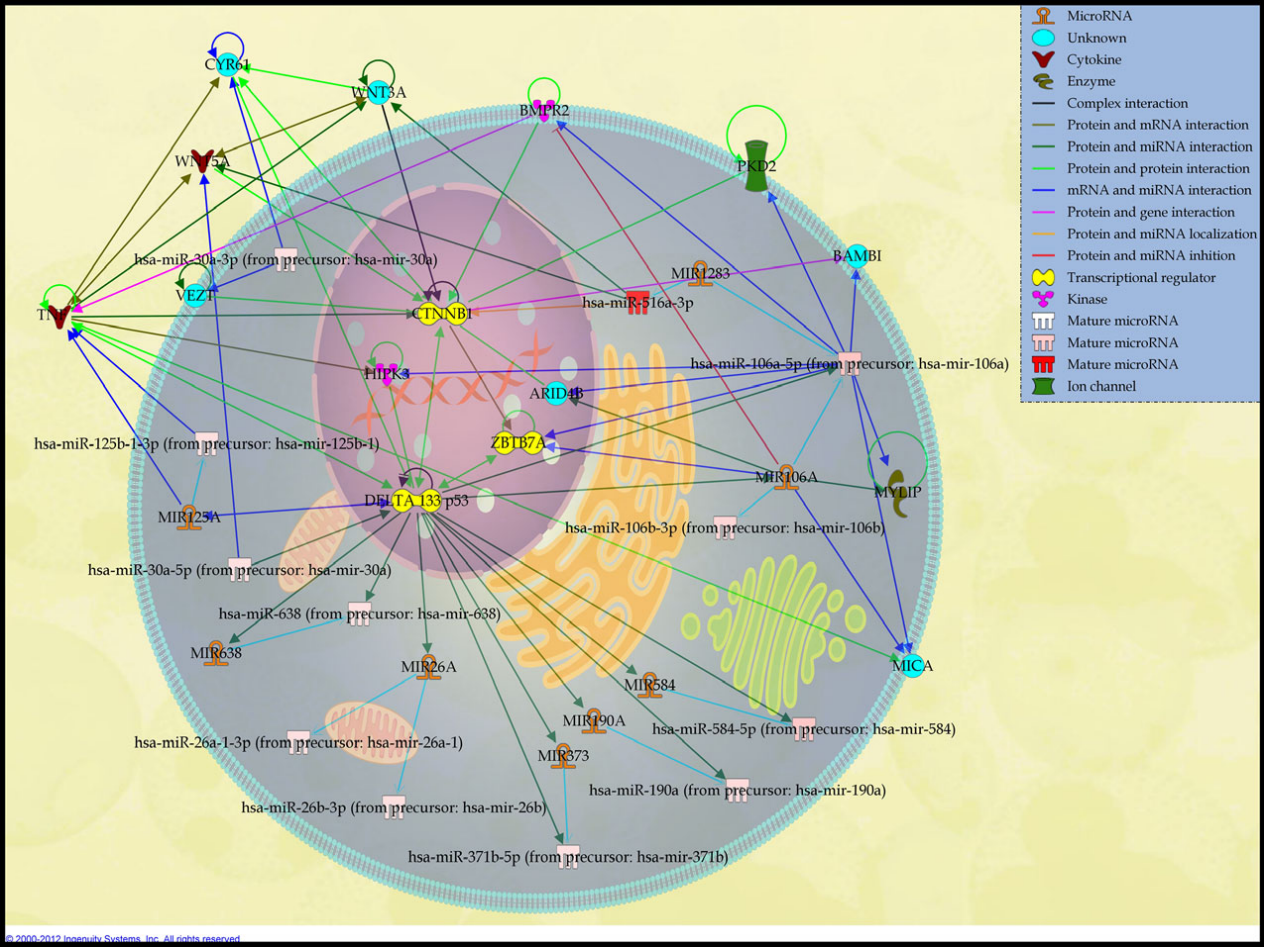
Network 1: Schematic representation describing the interaction between 5 highly expressed miRNAs found in DPSCs with their associated target mRNAs and cellular proteins related to cancer, reproductive system disease and genetic disorder. The miRNAs are namely hsa-miR-516a-3p, hsa-miR-125b, hsa-miR-190a, hsa-miR-106a and hsa-miR-584-5p. Network was constructed by using Ingenuity software based on expression relationships described in the literature. For miRNA analysis, the colour intensities (from pink to red) were correlated with fold change intensities, in which miRNAs overexpressed in functional analysis, are indicated in red.

The biological functions generated from the up-regulated miRNA demonstrated its involvement in various pathways with the top 5 in the reproductive system disease, cancer, infectious disease, organismal development, connective tissue disorders and genetic disorders, whereas the lowest 5 pathways are in cellular development, cellular growth and proliferation, skeletal and muscular disorders, respiratory system development and function as well as haematopoiesis (Fig.1C). Within this, the software analysis identified 25 associated network functions whereby a few pathways may involve in creating a network function. Amongst this, top 2 networks were further analysed based on the statistical significance (*P* < 0.01) and biological relevance. In the first network that comprises of 12 miRNA, only 5 miRNAs were up-regulated (Fig.2, Table2). Among this, hsa-miR-516a-3p was noted with highest expression level and this miRNA is reported to regulate WNT and CTNNB1 mRNAs, which play an important role in WNT pathways. Next is has-miR-106a (developed from has-miR-106b), which regulates a diverse range of mRNA related to transcription regulators such as ZBTB7A, enzyme such as MYLIP and kinase such as BMPR2, MICA and ARID4B. The next up-regulated miRNA is hsa-miR-125b-1-3p, which regulates inflammatory-related mRNA, TNF. Surprisingly, we found that 2 up-regulated miRNAs, hsa-miR-584-5p and miR-190a, were co-regulated by DELTA 133 p53 mRNA, which is known to be involved in tumourigenesis.

**Table 2:** Top two associated network functions generated by using Ingenuity Pathway Analysis

Network	miRNA	Abbr.	Entrez gene name	Function
Reproductive system disease, Cancer, Genetic disorder	miR-638	ARID4B	AT-rich interactive domain 4B (RBP1-like)	Other
	miR-26a-1-3p	BAMBI	BMP and activin membrane-bound	Other
	miR-294-5p	BMPR2	inhibitor homologue	Kinase
	miR-30c-5p/miR-30c/miR-30b-5p	CTNNB1	bone morphogenetic protein receptor, type II	Transcription
	miR-30a-3p/miR-30d-3p/miR-30e	CYR61	catenin (cadherin-associated protein),	Regulator
	miR-26b-3p/miR-26b^*^/miR-26a-2-3p	HIPK3	beta 1, 88 kD	Other
	miR-125b-1-3p/miR-125b-3p	MICA	cysteine-rich, angiogenic inducer, 61	Kinase
	miR-190a	MYLIP	homeodomain interacting protein kinase 3	Other
	miR-106a	PKD2	MHC class I polypeptide-related sequence A	Enzyme
	miR-584-5p	TNF	myosin regulatory light-chain interacting	Ion channel
	miR-17-5p/miR-20b-5p/miR-93-5p	VEZT	protein polycystic kidney disease 2	Cytokine
	miR-516a-3p/miR-516b-3p	WNT3A	(autosomal dominant)	Other
		WNT5A	tumour necrosis factor	Cytokine
		ZBTB7A	vezatin, adherens junctions transmembrane protein	Cytokine
			wingless-type MMTV integration site family, member 3A	Transcription regulator
			wingless-type MMTV integration site family, member 5A	
			zinc finger and BTB domain containing 7A	
Genetic disorder, Skeletal and muscular disorder, Developmental disorder	miR-543-3p/miR-543^*^/miR-543	DICER1	dicer 1, ribonuclease type III	Enzyme
	miR-409-3p (human, mouse)	EGFR	epidermal growth factor receptor	Kinase
	miR-409-5p	EIF2C2	eukaryotic translation initiation factor 2C, 2	Translation
	miR-4712-5p/miR-770-5p	FOS	FBJ murine osteosarcoma viral oncogene	regulator
	miR-425-3p/miR-425^*^	IRS1	homologue	Transcription factor
	miR-656	NR0B2	insulin receptor substrate 1	Enzyme
	miR-539		nuclear receptor subfamily 0, group B, member 2	Ligand dependent nuclear receptor
	miR-431			
	miR-495			
	miR-494			
	miR-487			
	miR-382			
	miR-7-5p/miR-7a-5p/miR-7a			
	miR-221-5p/miR-221^*^			
	miR-377-5p/miR-672-5p/miR-672			
	let-7f-2-3p			
	miR-376a-5p			

**Fig 3:**
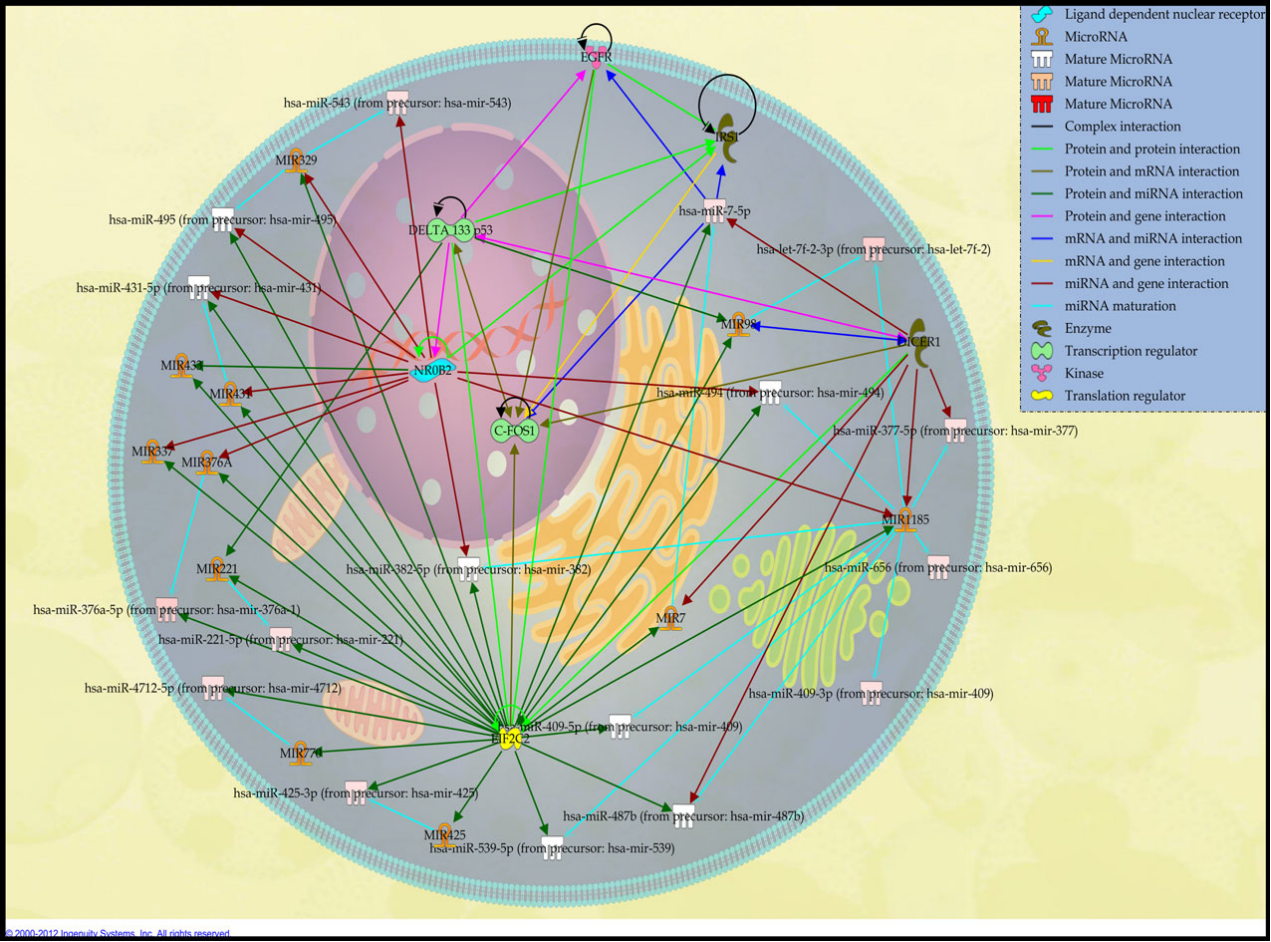
Network 2: Schematic representation describing the interaction between 5 highly expressed miRNAs found in DPSCs with their associated target mRNAs as well as cellular proteins related to genetic, developmental, skeletal and muscular disorder. The miRNAs are namely hsa-miR-7-5p, hsa-miR-221-5p, hsa-miR-377-5p, hsa-miR-376a-5p, and let-7f-2-3p. Network was constructed by using Ingenuity software based on expression relationships described in the literature. For miRNA analysis, the colour intensities (from pink to red) were correlated with fold change intensities, in which miRNAs overexpressed in functional analysis, are indicated in red.

A total of 5 up-regulated miRNAs of 16 miRNAs were noticed in 2nd network (Fig.3, Table2). Among these, hsa-miR-376a-5p and hsa-miR-221-5p were regulated by translation regulator-related mRNA, EIF2C2, while hsa-miR-377-5p directly regulated by DICER1. The most highly up-regulated miRNA is the hsa-miR-7-5p, which controls and acts on kinase-related gene EGFR, enzyme-related gene IRS1 and also transcriptional regulator C-FOS1. Apart from that, it is also shown that EIF2C2 and DICER1 modulate hsa-miR-7-5p. However, there were no possible targets for hsa-let-7f-2-3p.

### Validation of the differentially expressed miRNAs by using qRT-PCR

**Fig 4:**
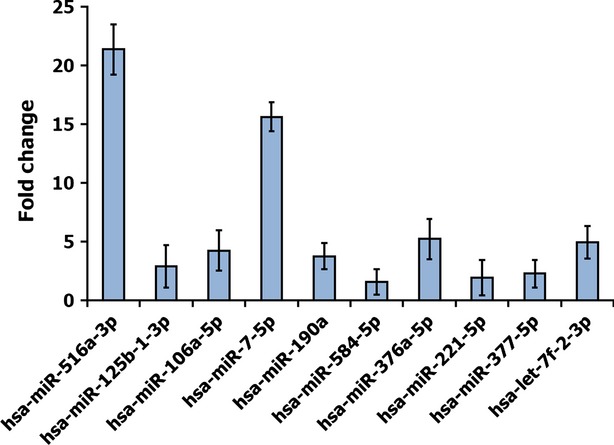
Validation of 10 highly expressed miRNAs in DPSCs using qRT-PCR. Generally, the higher a fold change value, the more copies are present in the specific sample. The miRNAs expression levels were calculated by using comparative cycle threshold (Ct) method. Ct values of target miRNAs were normalized in relation to U6 snRNA, which is an internal control gene. The fold change was calculated by using the equation 2^−ΔΔ^^CT^.

The distinct expressions of the 10 miRNAs found in DPSCs in both networks were further validated by using qRT-PCR analysis. The results for up-regulated miRNAs in DPSCs are shown in Figure4, which are significant relative to BM-MSCs. Consistent with the array results, the hsa-miR-516a-3p and hsa-miR-7-5p exhibited substantial increase in expression in DPSCs and further carried out in the downstream work.

### hsa-miR-516a-3p, the highly expressed microRNA, indirectly targets WNT5A gene

**Fig 5:**
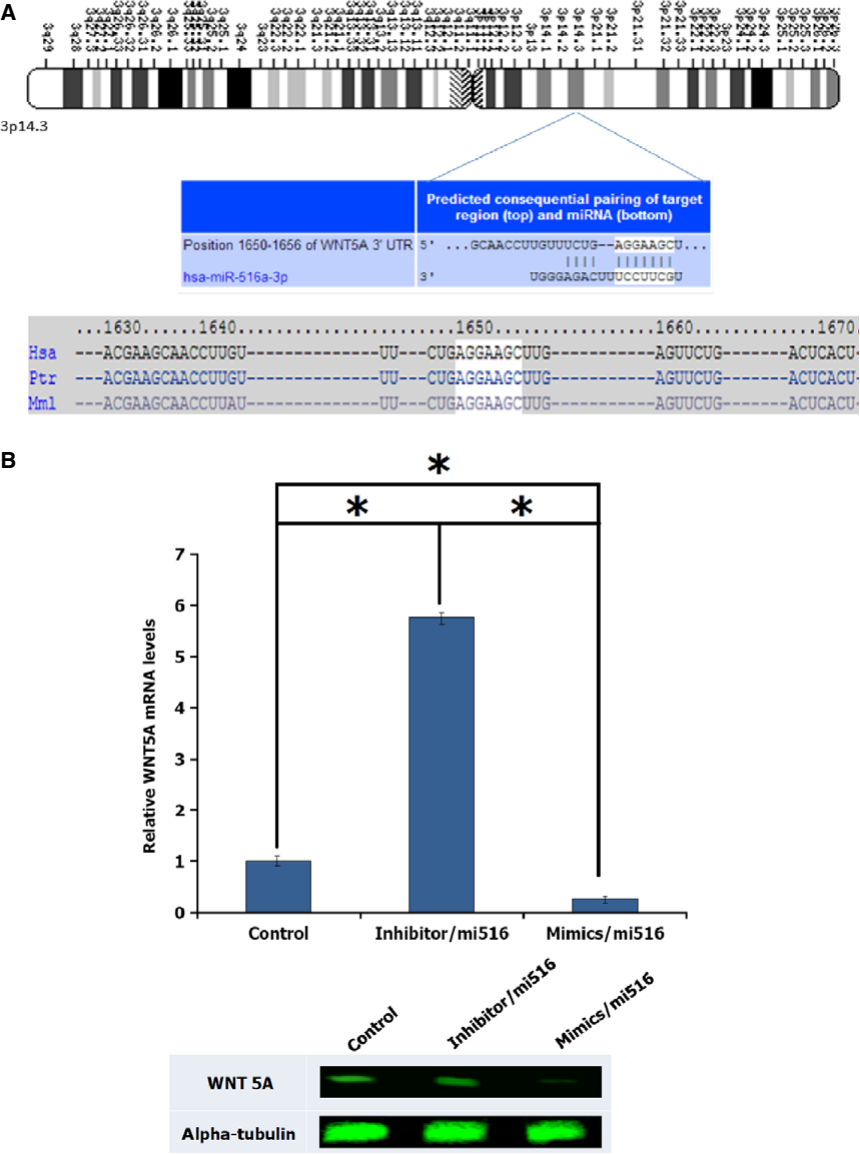
WNT5A is a potential hsa-miR-516a-3p target. (A) Sequence alignment of hsa-miR-516a-3p and predicted binding sites in the 3′-UTR of WNT5A (http://www.targetscan.org). (B) Quantification of WNT5A mRNA expression levels in response to the mimic and inhibitory effect of hsa-miR-516a-3p. (C) Protein level expression of the results shown in (B). Data are shown as the mean of SD values (*n* + 3).

To examine the role of miR-516a-3p in DPSCs, we focused our attention on elucidating the role of these microRNA on its target mRNA. Computational analysis indicated that WNT5A is a potential hsa-miR-516a-3p target because its 3′-UTR is matched to the hsa-miR-516a-3p seed region (Fig.5A). To investigate whether WNT5A is regulated post-transcriptionally, we examined the expression on mRNA and protein level by performing gain- or loss-function assay. In DPSCs, dramatic reduction of WNT5A mRNA was detected in overexpressed hsa-miR-516a-3p by qRT-PCR analysis as well as in the western blot result, while knockdown of has-miR-516a-3p enhanced their expression (Fig.5B).

**Fig 6:**
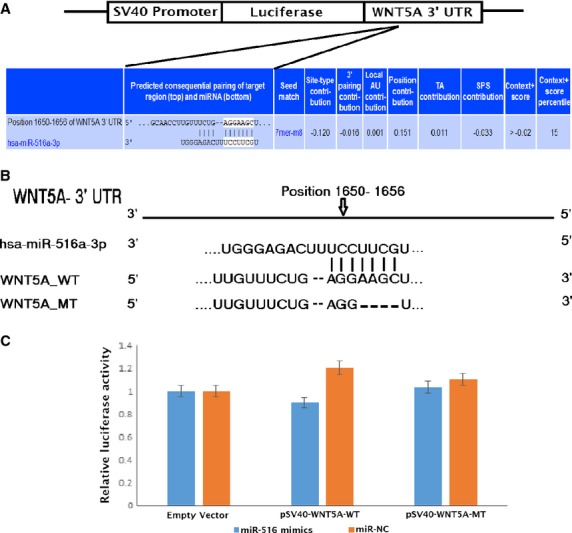
Validation of WNT5A gene as a target gene of hsa-miR-516a-3p. (A) Schematic diagram of luciferase reporter constructs for consensus hsa-miR-516a-3p target sites at the 3′-UTR region. (B) The sequence alignment of the predicted hsa-miR-516a-3p binding site in the 3′-UTR region of human WNT5A is shown with the seed target sequence (UCCUUCG). (C) Luciferase reporter vector containing hsa-miR-516a-3p target seed region of WNT5A (wild-type) or same vector without target seed region (empty vector) or same vector with deletion of the target seed region (mutant) were cotransfected with miR-516 mimics or negative control respectively. Data are representative of at least three technical experiments.

However, based on literature, Takei *et al*., 17 have debated that expression of WNT5A and WNT3A is indirectly regulated by hsa-miR-516a-3p because of other target, specifically SULF1 (extracellular sulfatase). Therefore, we proceeded to find the relative interaction between the hsa-miR-516a-3p and its predicted WNT5A mRNA 3′-UTR target sites by generating reporter vectors containing seed region complementarity to the miRNA upstream of the open reading frame (Fig.6A). These were constructed by using wild-type of WNT5A 3′-UTR sequences and the same sequence with four point mutation (deletion; Fig.6B). In DPSCs transfected with mimic miR-516, no effect on the expression of reporter was observed comparable to the DPSCs transfected with miR-NC (Fig.6C) impeding the specificity of the binding sequences. This finding is in agreement with the reported result by Takei *et al*.,^17^.

### EGFR gene as a direct target of microRNA hsa-miR-7-5p

**Fig 7:**
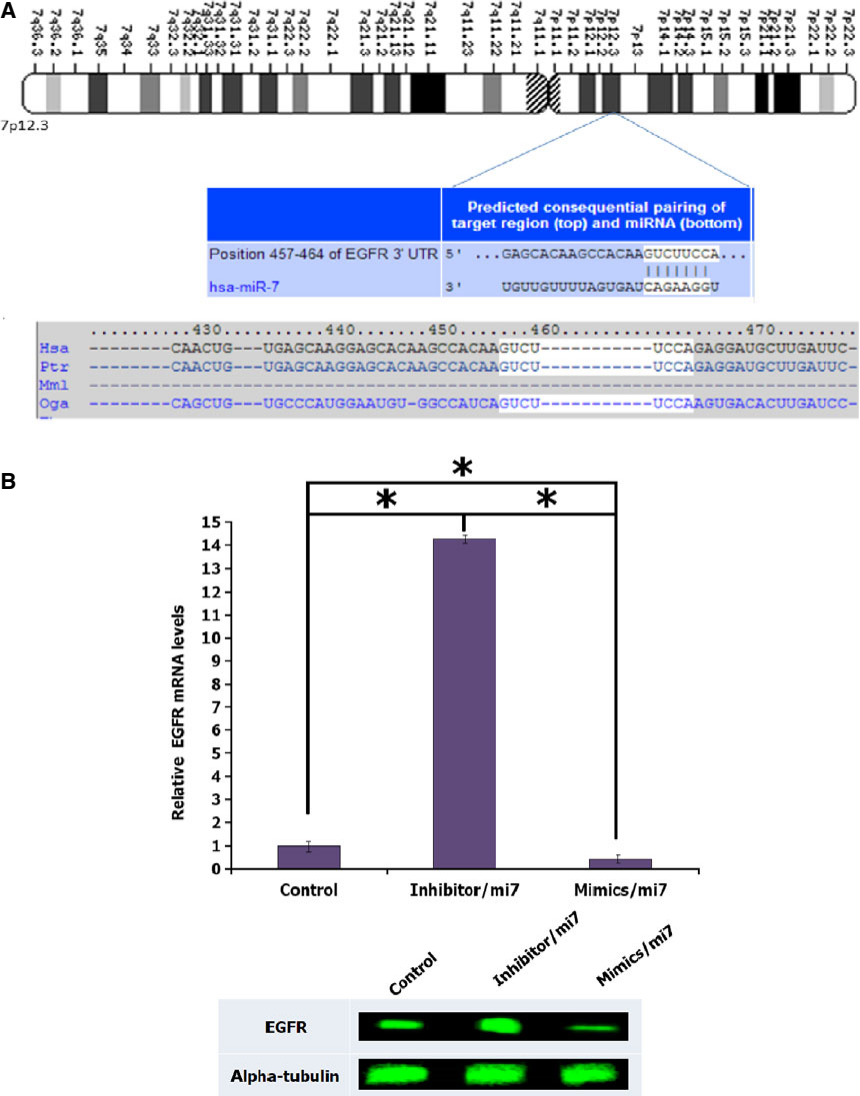
EGFR is a potential hsa-miR-7-5p target. (A) Sequence alignment of hsa-miR-7-5p and predicted binding sites in the 3′-UTR of EGFR (http://www.targetscan.org). (B) Quantification of EGFR mRNA expression levels in response to the mimic and inhibitory effect of hsa-miR-7-5p. (C) Protein level expression of the results shown in (B). Data are shown as the mean of SD values (*n* + 3).

Likewise, we found that EGFR is a potential hsa-miR-7-5p target as its 3'UTR is matched with hsa-miR-7-5p seed region (Fig.7A). Consistently, our results displayed that overexpression of the miRNA reduced mRNA and protein level of EGFR, while knockdown of the miRNA increased the mRNA as well as the protein level of EGFR (Fig.7B). Our results correspond well with previous works 18 conducted to validate the interaction between hsa-miR-7-5p and EGFR 3′-UTR target sites by using reporter assay. Hence, no further validation was carried out for hsa-miR-7-5p and EGFR target relation.

## Discussion

In the present study, DPSCs exhibited typical MSCs characteristics; fibroblastoid morphology, proliferation, multipotent differentiation capability and the expression of a typical set of surface protein. Nevertheless, variations were still noted between DPSCs and BM-MSCs and a key factor that attributes to this phenomenon probably because of its intrinsic molecular propensity that governs the fate of the cells. Various gene expressions are being controlled by miRNAs, and they also partly act in mutual negative feedback loops with protein factors to control cell fate decisions that are elicited by signal transduction activity. These findings implicate miRNAs as important mediators of gene regulation in response to cell–cell signalling 19. Dysregulation of these molecules often ends with an uncontrolled growth stage in the cell population 20. Hence, there is a need to identify miRNA activity in DPSCs to enable understanding of expression patterns that might be applicable prior to its usage in cell therapy.

Based on 2 network systems, 2 miRNAs were highly up-regulated and the remaining was moderate. Here, we briefly discuss the functions of each up-regulated miRNAs. An augmented level of hsa-miR-125b-1-3p expression was observed in the present study. This miRNA was involved in the regulation of TNF, a pro-inflammatory cytokine. This gene is said to function as critical feedback in signal molecules between immune cells and MSCs for MSCs-mediated immunosuppressive activities 21. Interestingly, there are also reports indicating that hsa-miR-125b-1-3p plays a vital role in suppressing osteogenic differentiation in MSCs 22. Furthermore, hsa-miR-125b-1-3p has been indicated to be involved in MSCs ageing, with a study reporting down-regulation of expression in MSCs in old primates compared with young primates 23. Similarly, the higher expression of hsa-miR-125-1-3p in DPSCs could be as a result of the fact that the cells were isolated at an early stage of adult tooth development 24. Overexpression of hsa-miR-125b-1-3p, which was done elsewhere, showed a significant increase in SC population, while depletion of the miRNA increased the non-SC population through WNT signalling 25. This was also reported by Lee *et al*. 26, who described that the depletion of human hsa-miR-125b-1-3p leads to the critical role of proliferation of differentiated cells.

Hsa-miR-106a-5p has been reported to target BMP groups, thus inhibiting the cells from osteogenesis 27. We believe that this miRNA down-regulates BMPR2, which is a kinase-related gene acting as potent inducer for osteogenesis differentiation and cell growth through Smad signalling 28. The computational data also predicted that hsa-miR-106a-5p is connected to various mRNAs such as ARID4B, ZBTB7A, BAMB1, PKD2, BMPR2, MYLIP and MICA. Among these mRNAs, ARID4B are cell cycle inhibitors 29, and the elevated expression of hsa-miR-106a-5p suggests that it enables cell proliferation in DPSCs. On the other hand, ZBTB7A, a transcription factor, regulates differentiation in multiple tissues and cell lineage, mainly oligodendrocyte lineage cells 30. However, in DPSCs, the hsa-miR-106a-5p could suppress the response of this gene and allow proliferation rather than differentiation. Apart from this, according to Shangguan *et al*. 31, increase in BAMB1 expression could block the differentiation of MSCs into carcinoma cells through TGF-β/Smad signalling in BM-MSCs. Besides that, BAMB1 are also a negative regulator for adipogenesis 32, while positively modulating WNT signalling 33 to promote cell cycle progression. In DPSCs, hsa-miR-106a-5p probably suppresses the BAMB1 and could instead play the antagonistic role. Hsa-miR-106a-5p also reacts to PKD2, which allows calcium influx 34 into cells that would trigger maturing of cells into specialized functions. Sun *et al*. 35 reported that miR-17 directly targets PKD2, and post-transcriptionally represses PKD2 expression, which leads to cell proliferation. Hence, in DPSCs, hsa-miR-106a-5p is predicted to suppress PKD2 to enable cell proliferation. The other gene that is regulated by hsa-miR-106a-5p is MICA, which is known to activate natural killer receptor and induce immune surveillance in cancer cells 36. One of the roles performed by MICA is to promote cytotoxic response during infection by binding with endothelial cells of the graft and induce cell destruction. The suppression of this immune-related gene in DPSCs by RNA interference may be used in transplantation, and also as a therapeutic target gene.

Hsa-mir-376a-5p has been known to suppress proliferation while inducing apoptosis in hepatocellular carcinoma cells 37. This miRNA, along with hsa-mir-377-5p, also known as chondro-miRs (with chondrocyte targets such as TGFbR, MAP3K, collagens, SMADs and cadherins), acts as a mediator of chondrogenic signalling pathways. This includes cell–cell interactions, TGF-β and MAPK signalling, which suggests a mechanism for genetic induction of chondrogenic differentiation 38. Along with that, hsa-miR-221-5p in DPSCs tends to inhibit osteogenesis in MSCs 39. Another miRNA, which is present in DPSCs, hsa-let-7f-2-3p, is known to be a pro-differentiation factor with ‘anti stemness’ properties 40. Our results predicted that translation factor EIF2C2 regulates these miRNAs. EIF2C2 is a short-interfering RNA that mediates gene silencing, which suggests its involvement in controlling lineage-restricted pathway 41. DICER-1 and EIF2C2 interact together to function as a translation initiation factor for short interfering RNA-mediated post-transcriptional gene silencing similar to the role played by miRNA. Bahubeshi *et al*., 42 demonstrated that DICER1 could function as the sole member of the miRNA pathway in which germline mutations induced the carrier to develop a human disease. Therefore, the functions carried out by the above miRNAs are suppressed because of the effects of DICER-1 and EIF2C2, bringing about the loss of chondrogenic differentiation or perhaps contributing to other lineage development. Hsa-miR-190a is one of the poorly characterized miRNAs. Previous study focused on the involvement of this miRNA in the development of tolerant to μ-opioid receptor agonists with NEUROD1 (a neural differentiation marker) as the direct target for mir-190 43. Likewise, hsa-miR-584 is known to play an important role in tumourigenesis process by inhibiting them 44.

We paid special attention to hsa-miR-516a-3p and hsa-miR-7-5p as these miRNAs were highly expressed upon validation with qRT-PCR analysis. We further proceeded with loss-of-function analysis with these miRNAs and we observed that hsa-miR-516a-3p knockdown induced a significant increase in the expression of WNT5A. This gene is involved in controlling cell fate decision by integral involvement in maintenance and growth 45. Furthermore, in tooth development, this gene is involved in regulating 4 cell signal pathways, namely JNK and AKT signal pathways as well as P42/44/MAPK and P38/MAPK pathways, which have close relation with cell proliferation and differentiation 46. Apart from this, we also predicted that hsa-miR-516a-3p down-regulates CTNNB1 gene, also known as beta-catenin, which is associated with WNT signalling for SCs renewal 47. WNT, together with Beta-catenin signalling pathway, represent a diverse group of molecules involved in controlling transcription of pluripotent genes, self-renewal and differentiation in most of the SCs found in adult tissues 48. Meanwhile, Blauwkamp *et al*. 49 reported that different levels of WNT signalling lead to distinct lineage-specific differentiation properties in human embryonic SCs. As in haematopoietic SCs, WNT signalling together with beta-catenin form a complex pathway which are shown to be more essential for development rather than for maintenance 50. Hence, we suggest that hsa-miR-516a-3p acts by suppressing the expression of WNT5A genes involved in WNT signalling pathway *via* altering/elevating SCs from undifferentiated to differentiated state. Nonetheless, it was puzzling to observe higher proliferation and less differentiation capacity in DPSCs when it was supposed to be the other way round. One of the possible reasons is the high expression of several pluripotency transcription factors such as Oct-4, Sox-2 and Nanog in DPSCs 16 with Wnt signalling pathway directly encompassing these genes. In addition, Oct 4 is involved in the maintenance of SCs fate *via* interaction with Wnt signalling pathway 51. Nevertheless, our further work to investigate the predicted interaction between the hsa-miR-516a-3p and mRNA WNT5A does not satisfy the criteria of miRNA and target prediction. This outcome corresponded to the work conducted by Takei *et al*., 17 that the WNT5A expression changes are most probably because of SULF1, which plays an important role in promoting WNT signalling pathway 52. Consistent with the above findings, Hayano *et al*.,^53^ have postulated that regulation of WNT signalling is modulated by SULF enzymes, which eventually control the differentiation of mouse pulp cells into odontoblasts. Our findings and notions warrant further investigation on the relationship between hsa-miR-516a-3p and WNT signalling pathway.

Another gene, EGFR, is known to suppress the osteoblast differentiation by inhibiting expression of transcription factors 54. As in neural SCs, the EGFR is known to promote cell number and self-regeneration 55. We found in this study that the positive interaction between hsa-miR-7-5p and its target EGFR through gain and loss assay. Validation of this interaction was found in a few studies that confirm that hsa-miR-7-5p directly targets EGFR 56,57. Therefore, we assume that in DPSCs, the role of EGFR is suppressed, which ultimately maintains SC numbers.

In addition to targeting EGFR, hsa-miR-7-5p targets upstream regulator, insulin receptor substrate (IRS-1) of the Akt pathway, which is essential for regulation of cell cycle progression, cell survival and cellular growth as noted by Kefas *et al*. 58. Besides that, hsa-miR-7-5p also down-regulates C-FOS1 transcription regulator, which is known as a marker for neuron activity. C-FOS1 is a member of AP-1 transcription factors that activate many genes, including those involved in cell growth and proliferation 59. Thus, hsa-miR-7-5p in DPSC is predicted to inhibit C-FOS1, eventually decreasing cell proliferation and growth. EGFR, IRS-1 and C-FOS-related genes are also connected to MAPK signalling pathway that regulates proliferation, gene expression, differentiation, mitosis, cell survival and apoptosis using a diverse range of stimuli 60. Therefore, we suggest that the role of hsa-miR-7-5p in gene regulation may suppress cell cycle progression and proliferation, either for differentiation or to maintain DPSCs in a quiescent state.

## Conclusion

In conclusion, our data suggest that miRNAs expressed in DPSCs preferentially express and integrate appropriately as a group, rather than playing a solitary role to create a functional switch between self-renewal, stemness and lineage development. These findings, along with further studies, can introduce a new dimension of gene regulation in controlling SC fate and behaviour in DPSCs, and facilitate development of therapeutic approaches for various diseases.

## Supporting Information

**Figure S1.** DPSCs exhibit favourable phenotype of MSCs.

**Figure S2.** Characterization of DPSCs by mesenchymal stem cell-related surface markers by FCM analysis.

**Figure S3.** DPSCs are bona fide MSCs based on multipotency ability.

**Figure S4.** Quantitative assessment on two highly expressed miRNAs; hsa-miR-516a-3p and hsa-miR-7-5p in DPSCs.

**Table S1.** The primer sequences for tri-lineage RT-PCR evaluation. **Table S2.** The product ID of the 10 miRNAs for validation by qRT-PCR. **Table S3.** Fold change value of differentially expressed miRNAs in DPSCs.
