# Supplementary figures and images for: Differential expression of basal microRNAs’ patterns in human dental pulp stem cells
Source: J Cell Mol Med. 2014 Dec 5;19(3):566–80. doi: 10.1111/jcmm.12381 (PMC4369814; doi:10.1111/jcmm.12381)

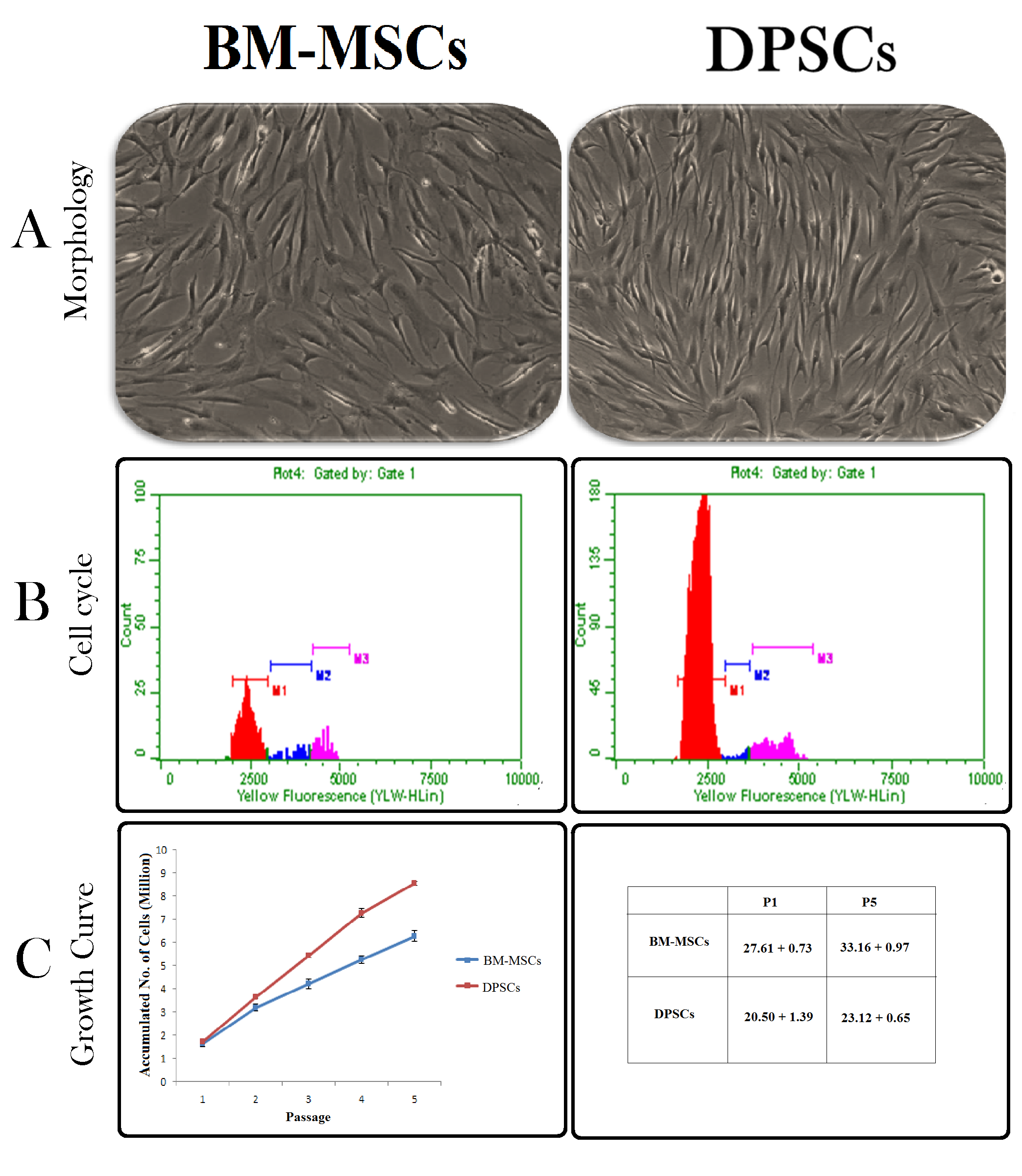

Supplement: Supplementary file 1 [file jcmm0019-0566-sd1.tif]

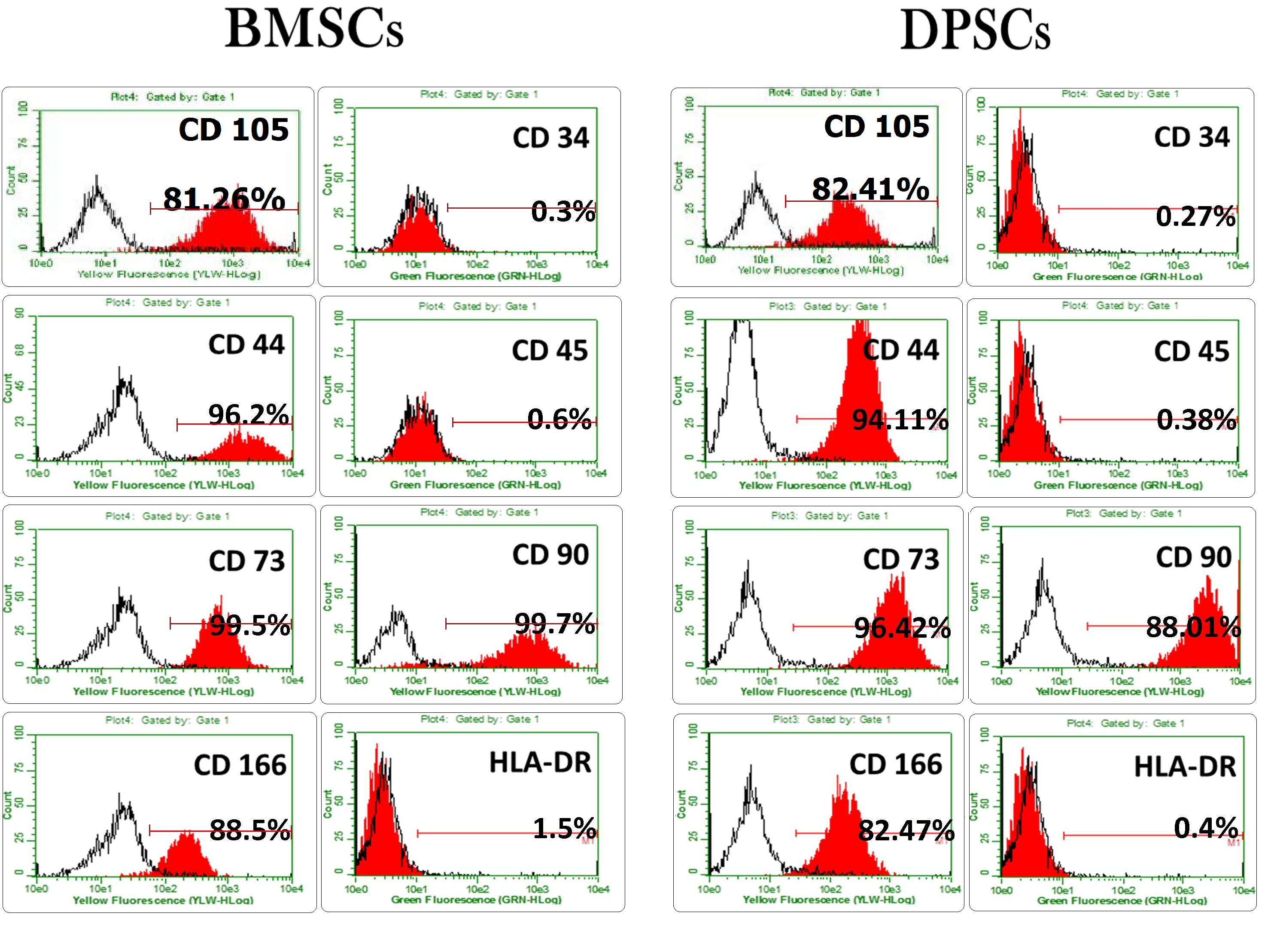

Supplement: Supplementary file 2 [file jcmm0019-0566-sd2.tif]

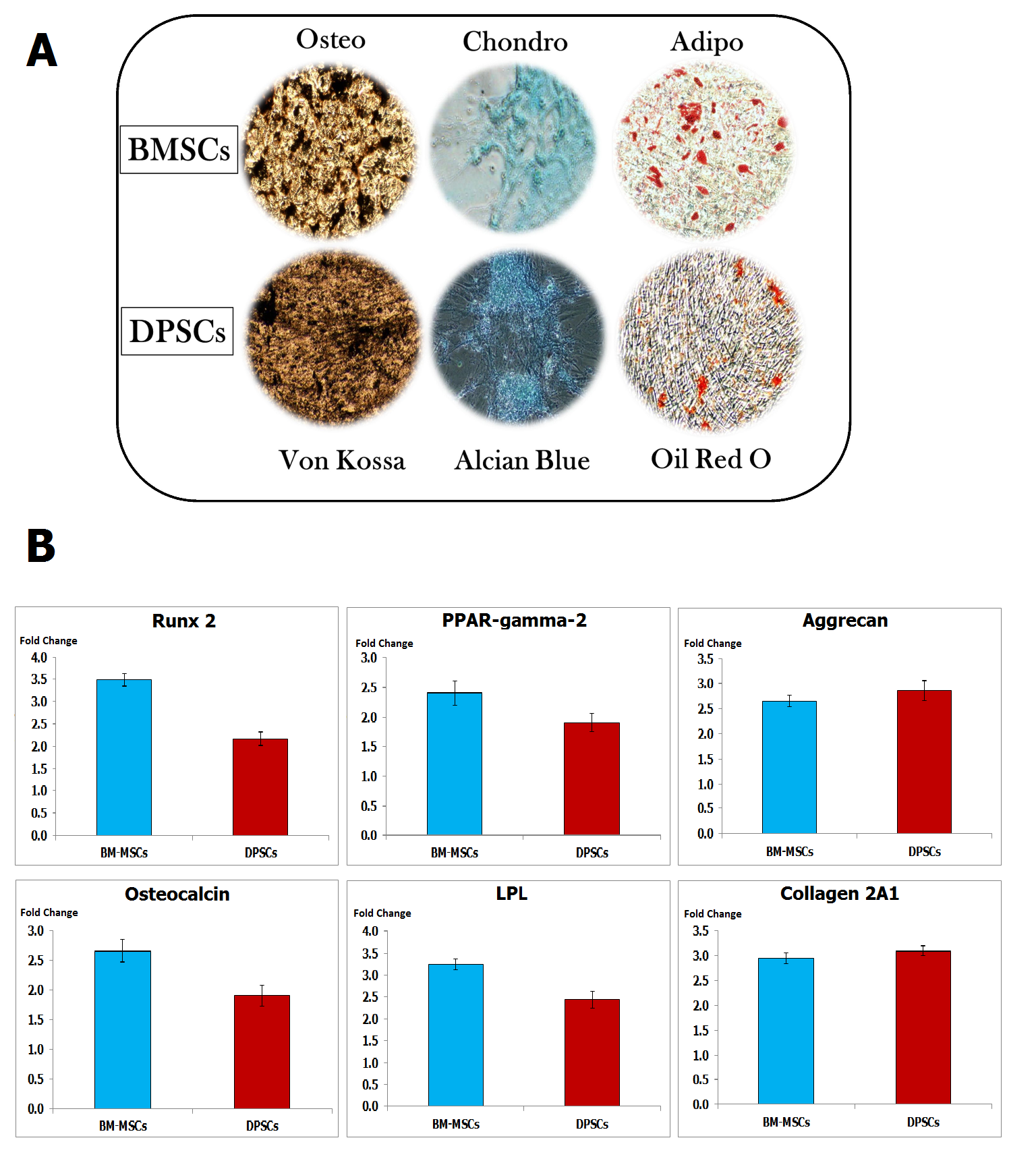

Supplement: Supplementary file 3 [file jcmm0019-0566-sd3.tif]

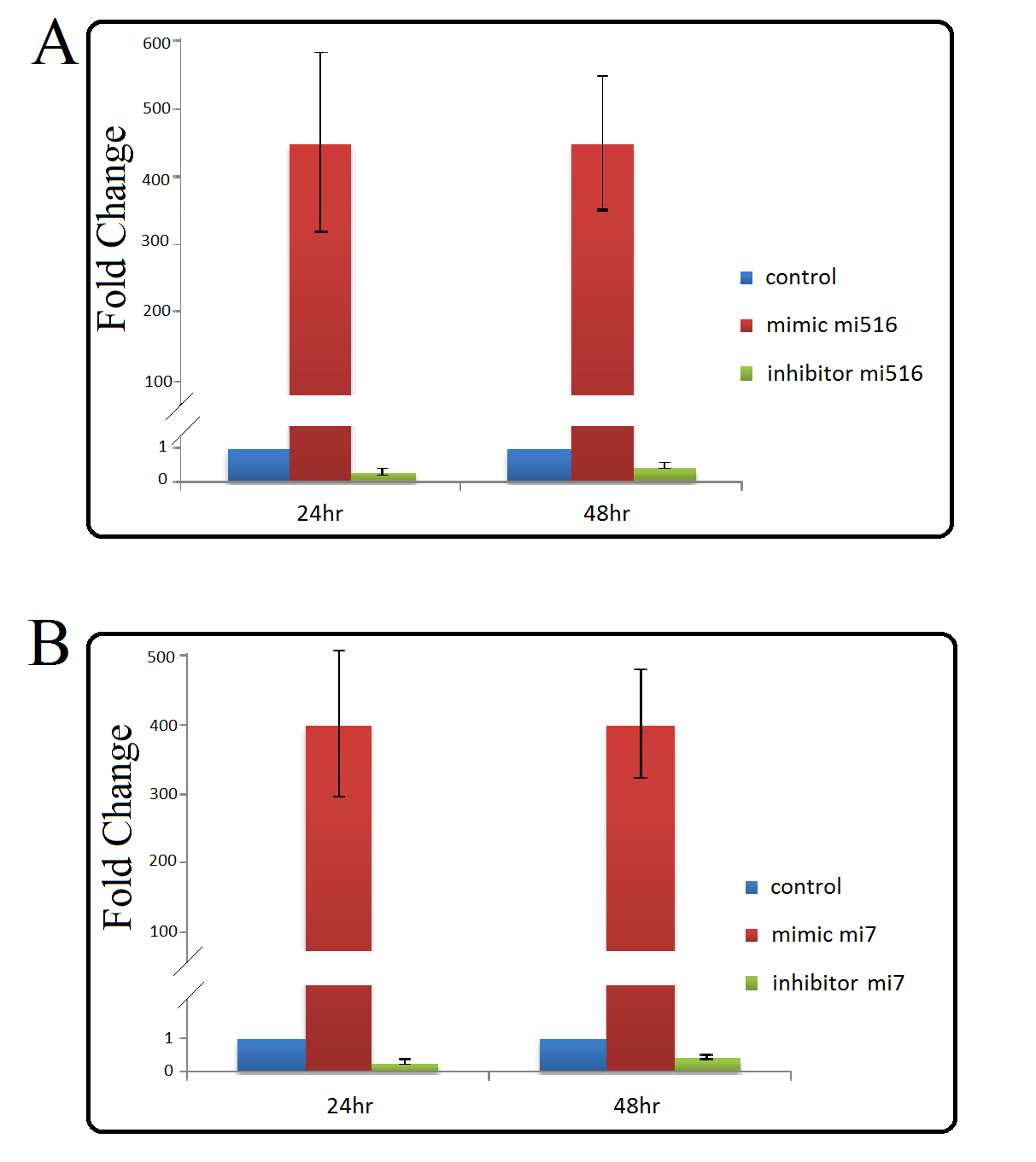

Supplement: Supplementary file 4 [file jcmm0019-0566-sd4.tif]
