# Supplementary material for: Differential expression of basal microRNAs’ patterns in human dental pulp stem cells
Source: J Cell Mol Med. 2014 Dec 5;19(3):566–80. doi: 10.1111/jcmm.12381 (PMC4369814; doi:10.1111/jcmm.12381)
Supplement: Supplementary file 5 [file jcmm0019-0566-sd5.docx]

Supplementary Table S1: The primer sequences for tri-lineage RT-PCR evaluation

| **Target gene product** | **Primer sequence** | **Annealing temperature** |
| --- | --- | --- |
| Runt-related transcription factor 2 (RUNX2) | 5′ CAGTGACACCATGTCAGCAA-3’  5′ GCTCACGTCGCRCATTTTG-3’ | 59°C |
| Osteocalcin (OST) | 5′-CATGAGAAGCCCTCACA-3’  5′-AGAGCGACACCCTAGAC-3′ | 55°C |
| Peroxisome proliferation activated receptor ɣ 2 (PPAR-ɣ 2) | 5′-TTCAGAAATGCCTTGCAGTG-3′  5′-GGGCTCCATAAAGTCACCAA-3′ | 57°C |
| Lipoprotein lipase (LPL) | 5′-TCAATCACAGCAGCAAAACC-3′  5′-CCACATCTCCAAGTCCTCTC-3′ | 57°C |
| Aggrecan (AGC) | 5′-TCAGGAGGGCTGGAACAAGTACC-3′  5′-GGAGGTGGTAATTGCAGGGAACA-3′ | 57°C |
| Collagen 2A1 (COL2A1) | 5′-TTTCCCAGGTCAAGATGGTC-3′  5′-CTTCAGCACCTGTCTCACCA-3′ | 55°C |
| wingless-type MMTV integration site family, member 3A  (WNT3A) | 5’-TGGCTCCTCTCGGATACCTC-3’  5’-AAAGCTACTCCAGCGGAGGC-3’ | 60°C |
| wingless-type MMTV integration site family, member 5A  (WNT5A) | 5’-CTGGAGGTGCCATGTCTTCC-3’  5’-TCGGCTGCCTATTTGCATCA-3’ | 60°C |
| Alpha-Tubulin | 5`-GGGTGGGGGAGAGATTACCT-3`  5`- GCACATAGGCGGATTCCTGT-3` | 60°C |
| glyceraldehyde-3-phosphate dehydrogenase (GAPDH) | 5’- AGC CAC ATC GCT CAG ACA CC-3’  5’-GTA CGC AGC GGC CAG CAT-3’ | 55°C |

Supplementary Table S2: The product ID of the 10 miRNAs for validation by qRT-PCR

| **Target miRNA product** | **miRBase Accession Number** | **Mature miRNA Sequence** |
| --- | --- | --- |
| hsa-miR-516a-3p | MI0003180 | UGCUUCCUUUCAGAGGGU |
| hsa-miR-125b-1-3p | MI0000446 | ACGGGUUAGGCUCUUGGGAGCU |
| hsa-miR-106a-5p | MI0000113 | AAAAGUGCUUACAGUGCAGGUAG |
| hsa-miR-7-5p | MI0000263 | UGGAAGACUAGUGAUUUUGUUGU |
| hsa-miR-190a | MI0000486 | UGAUAUGUUUGAUAUAUUAGGU |
| hsa-miR-584-5p | MI0003591 | UUAUGGUUUGCCUGGGACUGAG |
| hsa-miR-376a-5p | MI0000784 | GUAGAUUCUCCUUCUAUGAGUA |
| hsa-miR-221-5p | MI0000298 | ACCUGGCAUACAAUGUAGAUUU |
| hsa-miR-377-5p | MI0000785 | AGAGGUUGCCCUUGGUGAAUUC |
| hsa-let-7f-2-3p | MI0000068 | CUAUACAGUCUACUGUCUUUCC |

Supplementary Table S3: Fold change value of differentially expressed miRNAs in DPSCs

| **Upregulated** | |  |  | **Downregulated** | |  |  |
| --- | --- | --- | --- | --- | --- | --- | --- |
| **miRNA** | **Fold Change** | **miRNA** | **Fold change** | **miRNA** | **Fold Change** | **miRNA** | **Fold Change** |
| hsa-miR-516a-3p | 22.743 | hsa-miR-30e* | 1.772 | hsa-let-7i* | 0.968 | hsa-miR-454* | 0.431 |
| hsa-miR-7 | 15.568 | hsa-miR-30a* | 1.361 | hsa-miR-100* | 0.939 | hsa-miR-513-3p | 0.426 |
| RNU43 | 10.362 | hsa-miR-30d | 1.016 | hsa-miR-30a | 0.898 | hsa-miR-29c* | 0.417 |
| hsa-miR-526b* | 7.629 | hsa-miR-30e | 1.539 | hsa-miR-188-5p | 0.857 | hsa-miR-16-1* | 0.415 |
| hsa-miR-376a* | 6.801 | hsa-miR-34a* | 1.769 | hsa-miR-214* | 0.855 | hsa-miR-941 | 0.414 |
| hsa-let-7f-2* | 5.421 | hsa-miR-411* | 1.786 | hsa-miR-432* | 0.84 | hsa-miR-432 | 0.398 |
| hsa-miR-106a-5p | 4.88 | hsa-miR-409-3p | 1.049 | hsa-miR-130b* | 0.82 | hsa-miR-136* | 0.397 |
| hsa-miR-190a | 4.505 | hsa-miR-424* | 1.377 | RNU48 | 0.797 | hsa-miR-661 | 0.367 |
| hsa-miR-378 | 4.132 | hsa-miR-425* | 1.157 | hsa-miR-93* | 0.762 | hsa-miR-99a* | 0.362 |
| hsa-miR-125b-1* | 3.708 | hsa-miR-770-5p | 1.135 | hsa-miR-7-1* | 0.671 | hsa-miR-520c-3p | 0.346 |
| hsa-miR-629* | 3.277 | hsa-miR-509-3p | 1.125 | hsa-miR-505* | 0.657 | hsa-miR-99b* | 0.346 |
| hsa-miR-939 | 3.317 | hsa-miR-601 | 1.486 | hsa-miR-181a* | 0.615 | hsa-miR-206 | 0.326 |
| hsa-miR-377* | 2.928 | hsa-miR-543 | 1.015 | hsa-miR-222* | 0.587 | hsa-miR-154* | 0.212 |
| hsa-miR-565 | 2.629 | hsa-miR-589* | 1.142 | hsa-miR-135a* | 0.58 | hsa-miR-630 | 0.202 |
| hsa-miR-766 | 2.593 | hsa-miR-625* | 1.095 | hsa-miR-493* | 0.578 | hsa-miR-379* | 0.198 |
| hsa-miR-148b* | 2.495 | hsa-miR-638 | 1.103 | hsa-miR-145* | 0.573 | hsa-miR-335* | 0.195 |
| hsa-miR-221* | 2.476 | hsa-miR-643 | 1.864 | hsa-miR-875-5p | 0.553 | hsa-miR-923 | 0.194 |
| hsa-miR-584 | 2.316 | hsa-miR-656 | 1.357 | hsa-miR-30d* | 0.547 | hsa-miR-550 | 0.19 |
| hsa-miR-564 | 2.001 | hsa-miR-769-5p | 1.227 | hsa-miR-550* | 0.534 | hsa-miR-10b* | 0.117 |
| hsa-miR-15b* | 1.798 | hsa-miR-877 | 1.701 | hsa-miR-21* | 0.531 | hsa-miR-18a* | 0.11 |
| hsa-miR-138-1* | 1.621 | hsa-miR-942 | 1.851 | hsa-miR-760 | 0.526 | hsa-miR-15a* | 0.063 |
| hsa-miR-149* | 1.649 | RNU24 | 1.658 | hsa-miR-20a* | 0.487 | hsa-miR-500* | 0.014 |
| hsa-miR-151-3p | 1.179 | RNU44 | 1.074 | hsa-miR-659 | 0.465 |  |  |
| hsa-miR-19b-1* | 1.48 | RNU6B | 1.675 | hsa-miR-126* | 0.456 |  |  |
| hsa-miR-27b* | 1.782 | hsa-miR-768-3p | 1.044 | hsa-miR-181a-2* | 0.454 |  |  |
| hsa-miR-22* | 1.123 | hsa-miR-373* | 1.441 | hsa-miR-801 | 0.454 |  |  |
| hsa-miR-26a-1* | 1.129 |  |  | hsa-miR-34b* | 0.439 |  |  |
| hsa-miR-26b* | 1.811 |  |  | hsa-miR-27a* | 0.434 |  |  |
